# The Spatial and Temporal Construction of Confidence in the Visual Scene

**DOI:** 10.1371/journal.pone.0004909

**Published:** 2009-03-17

**Authors:** Martin Graziano, Mariano Sigman

**Affiliations:** Integrative Neuroscience Laboratory, Physics Department, Universidad de Buenos Aires, Buenos Aires, Argentina; University of Minnesota, United States of America

## Abstract

Human subjects can report many items of a cluttered field a few hundred milliseconds after stimulus presentation. This memory decays rapidly and after a second only 3 or 4 items can be stored in working memory. Here we compared the dynamics of objective performance with a measure of subjective report and we observed that 1) Objective performance beyond explicit subjective reports (blindsight) was significantly more pronounced within a short temporal interval and within specific locations of the visual field which were robust across sessions 2) High confidence errors (false beliefs) were largely confined to a small spatial window neighboring the cue. The size of this window did not change in time 3) Subjective confidence showed a moderate but consistent decrease with time, independent of all other experimental factors. Our study allowed us to asses quantitatively the temporal and spatial access to an objective response and to subjective reports.

## Introduction

A vast ensemble of stimuli are continuously being processed in parallel by the sensory system, most of which elicit only a brief transient sensory response which fades after few hundred milliseconds without reaching working memory, executive control and consciousness [1], [2]. Determining what subset of this ensemble accesses awareness and what determines this access has been, in the course of the last years, a matter of intensive research. A consistent qualitative observation is that the subset of conscious information is significantly smaller than what would be inferred either by direct introspection - as demonstrated for instance in change blindness experiments - and by explicit behavioral measures - as demonstrated for instance in subliminal priming experiments [3]–[7]. Recently, several studies have shown that introspective measures are highly reliable and thus that understanding which aspects of information processing are accessible to introspection and which are opaque can be determined with accurate quantitative precision, a methodology referred as quantitative introspection [8]–[11].

Since the early experiments of Sperling [12], the *partial report paradigm*, has been used to understand the dynamics of information available for executive control and working memory. Sperling showed that when observers saw briefly presented displays composed of several alphanumeric characters, after a second only a few (3 to 5) were available for working memory. However, observers had a much better memory when required to identify a specific subset of the characters at an interval (Inter Stimulus Interval, ISI) after the presentation of the visual display. This indicated the existence of a high capacity initial memory of the stimulus display which decayed a few hundred milliseconds after stimulus presentation, referred as Iconic Memory [13].

Here we perform a partial report experiment in which, in addition, subjects reported the subjective confidence in their response, as a direct measure of the conscious access to the responded letter. We found a marked double dissociation between objective response and subjective confidence: instances in which subjects systematically responded correctly at very low confidence and others in which subjects responded systematically incorrectly with very high confidence in their response. These dissociations followed a well determined dependence with temporal and spatial properties of the stimuli, which allowed us to asses quantitatively the temporal course of the elements of the visual scene available for an objective response, and to consciousness.

## Results

### 1- Experimental design and reliability of objective and subjective measurements

**Figure 1:**
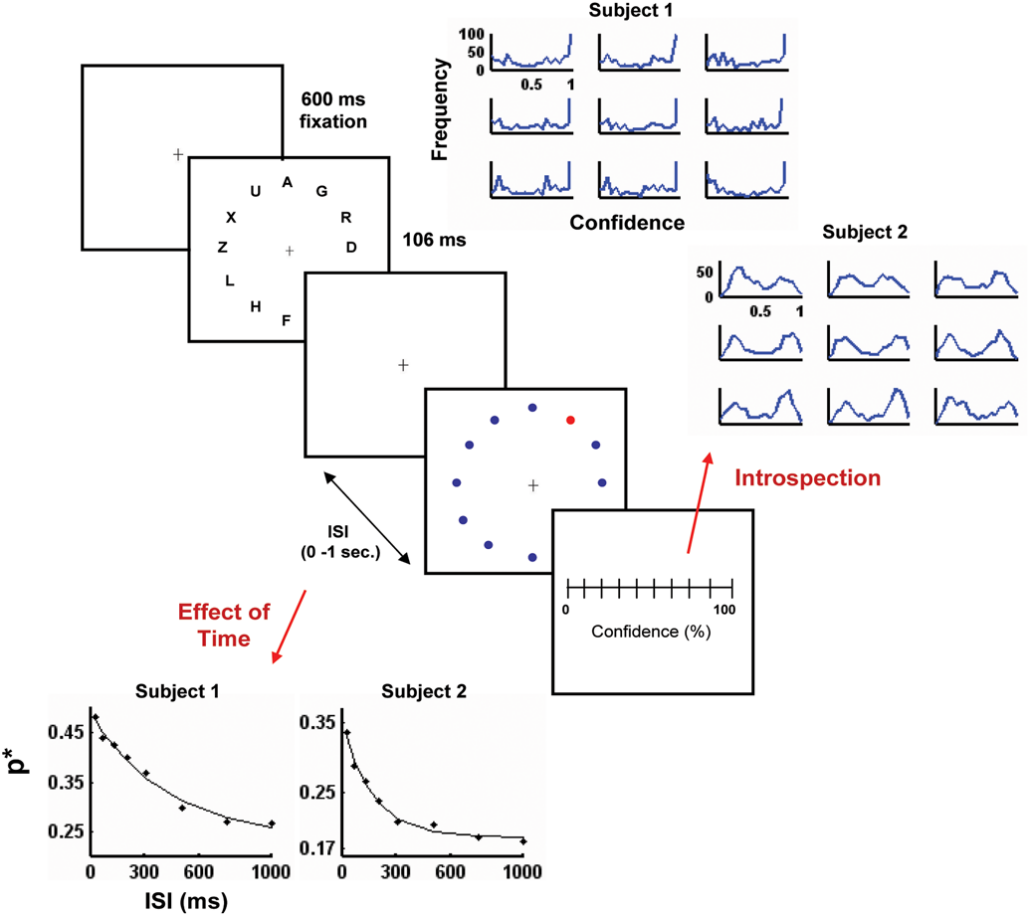
Experimental Design and reliability of measurements. A circular array of twelve letters was presented during 106 ms. Participants fixated in a cross at the center of the array. After a delay (which varied randomly between 0 and 1 s), a small red circle (the cue) was presented in one of the locations of the array indicating the letter that had to be responded. Then, participants had to report with the mouse the confidence level of their response through an *ad hoc* bar placed at the center of the screen. The response ranged between 0% of Confidence (guessing) and 100% (completely certain). The distribution of confidence reports are shown for every session of each subject at the Top-Right, showing a very high reliability across sessions. Both subjects showed a robust exponential decay of performance with ISI (Bottom-Left).

In each trial participants saw – while maintaining fixation in a cross at the center of the display - a circular twelve-letter array which lasted 106 ms (Figure 1). At a variable ISI, ranging from 24 to 1000 ms following the stimulus presentation, a small red circle was presented adjacent to a random location of the array which indicated the position of the letter that had to be responded. The cue was very small (12 times smaller than the average letter size) and placed at a larger eccentricity than the stimuli to minimize the possibility that it may induce masking of the target letters. The cue remained visible until subjects responded. To assure that subjects knew precisely the location of the cue, we performed a control experiment (see methods, Cue Position Control Experiment). The stimulus display was exactly as in the original experiment (fixation, array of letters, cue). After completion of the trial, the screen disappeared and after 1 sec, the subject was shown an array with all the locations and asked to report position of the cue. Performance was 100% in this control experiment indicating that subjects have perfect knowledge of the position of the cue.

Participants responded using a standard computer keyboard. Following this initial report, participants had to report the confidence level of their response with an *ad hoc* bar placed at the center of the screen (Figure 1). Participants indicated their confidence level in percent ratings between 0% of confidence - when they thought they were simply guessing - and 100% - when they were completely certain of their response. Two participants performed 9 experimental sessions (of 576 trials each) in different days. Participants performance decayed with ISI - as has been systematically reported in previous partial report paradigm experiments [12]. This decay could be observed in every individual session for both participants, and the average dependence of the performance with ISI could be fitted accurately by an exponential function - 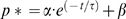
- [14] for each individual subject (Subject 1, α: 0.25±0.01, β: 0.24±0.01, τ: 411±33 ms, R^2^: 0.99; Subject 2, α: 0.17±0.01, β: 0.19±0.01, τ: 167±8 ms, R^2^: 0.99).

The measure of confidence was surprisingly reliable across different experimental sessions (Figure 1), almost determining a fingerprint of each individual subject. Indeed, the distributions were quite distinct for each subject (although roughly both were bimodal with a minimum in the intermediate confidence values) but, for each subject, this pattern was very reproducible across the different sessions.

These results simply show that participants performed accurately both in the objective and subjective tasks and thus that this data was reliable to study quantitatively the interaction between the content of iconic memory – as measured by explicit and subjective reports.

### 2- Seeing and believing: Sources of information for objective reports and subjective confidence

**Figure 2:**
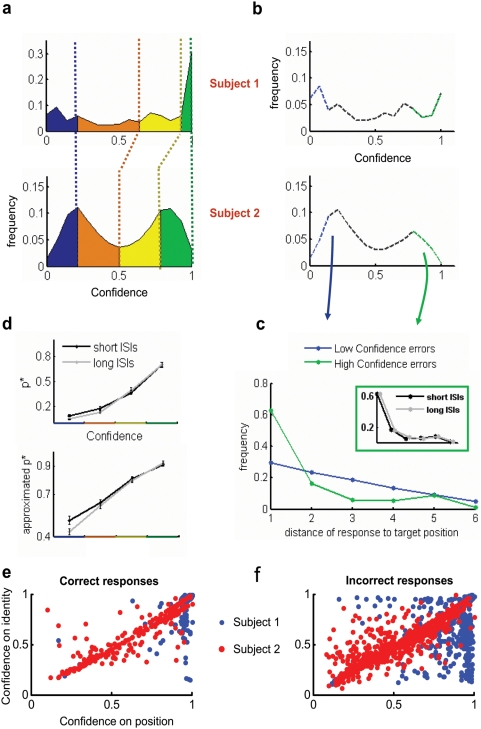
Correlations and Dissociations between objective and subjective reports. A) Confidence reports were grouped in four percentile groups of confidence (25, 50, 75 and 100% - blue, orange, yellow and green traces) for each individual session and participant. B) Subjective confidence distribution for incorrect responses (black dotted line). The blue and green traces indicate the low-confidence and high-confidence error trials. C) Distance between the responded letter and the cue when the responded letter was a distractor for high- confidence (green) and low-confidence (blue) errors. The inset shows that this distribution does not change for short and long ISIs. D) Mean performance increased with subject's confidence report. At low confidence, responses were more accurate for short ISI values. E and F) Control experiment in which subjects reported in two subsequent screens the confidence in the position of the seen letter relative to the cue and of the identity of the seen letter for correct (E) and error (F) trials.

To quantify the relation between objective performance (accuracy of the response) and participant's subjective confidence report, we binned the distribution of confidence report in four percentile groups (25, 50, 75 and 100%), for each individual session and participant (Figure 2A). Objective performance strongly correlated with subjective confidence report (Figure 2D). An ANOVA analysis showed a very significant effect of subjective confidence on performance (F_3, 8.13_ = 47.45, P = 0.0026).

To explore possible dissociations between objective and subjective measurements, we explored the distribution of subjective confidence reports for error trials (Figure 2B). This distribution, while biased to low confidence scores, shows for both participants a bimodal distribution with a shape very similar to the one observed in the distribution confidence for all (correct and error) trials (Figure 2A). This indicates the existence of a substantial amount of trials in which participant's response is incorrect and yet they are very certain about their response. We refer to the error trials in which the subjective confidence score is within the highest 25% percentile as *high-confidence errors* (green trace) and similarly as *low-confidence errors* to the error trials in which the subjective confidence score is in the lowest 25% percentile (blue trace).

To understand the mechanisms which may lead to the paradoxical *high-confidence errors*, we explored whether these trials may result from the incorrect localization of an object whose identity has been identified correctly, i.e. whether participants are reporting the letter of a distractor which was present in the array but not in the cued location. We found that in a very large fraction of error trials (Subject 1, 77.15±0.03%; Subject 2, 91.77±0.04%) participants responded a letter which was present in the array (chance level is at 44%). To further explore whether this errors may be clustered in space, we measured the distance between the position in the array of the responded letter and the position of the cue (Figure 2C). For this analysis we considered only the fraction of trials in which the responded letter was presented in the array and was not in the cued position. In these trials, the distance – described in number of elements of the array - varies from 1 (immediate neighbor) to 6 (antipode). In correct trials – which are not considered for this analysis - the distance is zero. We observed that *high-confidence errors* involve mostly responses of letters adjacent to the cued location and that the distribution of distance reaches a plateau at a distance of 3 elements which corresponds to an angle of 90 degrees, or simply a quadrant in the visual scene. The fraction of *low-confidence errors*, on the contrary, showed a moderate and progressive decrease with distance. The distance distribution for *high-confidence errors* did not changed with time (for short and long ISIs, inset Figure 2C).

These results indicate that participants may correctly identify a letter, yet misattribute the location –within a relatively fixed and bounded spatial window and independently of ISI. More importantly, our results show that this misattribution is insufficient to flag the error monitoring system [15], i.e. subjects were not aware of the fact that they have made a mistake.

To further explore whether the spatial misattribution leading to an error remains inaccessible to consciousness, we performed a new experiment [see methods, Feedback Control Experiment] in which we provided feedback to subjects in error trials in which the responded letter was at a distance smaller than 3 from the target. In these trials the subject was informed that he or she had made an error and was asked whether the responded letter was clockwise or anticlockwise from the cue. The results (performance - Subject 1: 49±5%, Subject 2: 43±4%) showed that subjects were completely at chance indicating that even after an error had been flagged, and subjects had responded to a close letter, they could not report the spatial direction of the spatial miss-location.

Finally, we wanted to control whether the miss- location between the responded letter and the cue involved the interaction and competition (scrambling) of multiple letters in the cluttered field or simply a drift in the position of the letter relative to the cue. To address this issue we performed an additional control experiment [see methods, Dot Probe Experiment]. The design was identical to the main experiment, except that during the display of the letters a small dot probe was presented on the inside of the ring of letters. Subjects were then asked to perform a dual-task: first to report the letter in the cued-location (as in the original experiment) and then to report whether the dot was clockwise or anti-clockwise respect to the cue. Performance in the location task was virtually perfect in this experiment (>95% for both subjects, a total of 120 trials) indicating that subjects can remember precisely and without drift a single location during the experiment and thus that location miss-attributions result from a complex interaction in space and time of the multiple elements presented in the array.

Based on these results, in what follows we also studied *approximate responses* (see for example,[16]), considering that a response is approximately correct if subjects respond to a letter which was presented at a distance shorter than three from the cued location. This is as if the cue would not be considered a focal point but rather a diffuse region in space which includes the neighboring letters [17].

### 3- Correlations and dissociations between objective performance and subjective confidence reports

Next we investigated whether experimental manipulations may dissociate the objective performance and participant's subjective confidence report, by studying objective performance at a fixed value of the subjective confidence score as a function of the critical experimental variable – the ISI (Figure 2D). From simple inspection of the curve, it can be seen that for low confidence values, performance is better at *short* ISI (<130 ms) than at *long* ISI (>500 ms). This difference vanishes for high confidence responses. An ANOVA analysis revealed that the interaction between ISI and Confidence was marginally significant (F_3, 83_ = 2.16, P = 0.0987), while the main effect of ISI was not significant (F_1, 0.92_ = 0.96, P = 0.52). As mentioned in the previous section, the main effect of Confidence in performance was highly significant (F_3, 8.13_ = 47.45, P = 0.0026). We then explored the effect of ISI and Confidence and their interaction on *approximate* objective performance. Similarly to what we had observed in the analysis of exact responses, we found an increase in approximate performance for short ISI only for low values of confidence. This effect was comparatively more pronounced than for exact responses and an ANOVA analysis revealed a significant interaction between ISI and Confidence (F_3, 83_ = 4.63, P = 0.0048). A *post-hoc* Bonferroni Test comparing performance at short and long ISI for the lowest confidence level shows a significant difference (p<<0.05), indicating and effect of ISI at this confidence level.

This finding indicates that for short ISI values, a fraction of the iconic buffer which includes a coarse spatial region covering the cue may be accessible to bias the response, without affecting subjective confidence. If subjective confidence could be proven to be indicative of target visibility this result would imply that blindsight - or the ability to rout information from the sensory to the decision making machinery in the absence of consciousness - decreases substantially after a few hundred milliseconds. However, subjective confidence is a complex construct and it is likely that different situations – not necessarily directly related to the absence of target consciousness - may result in a low subjective confidence estimate. For instance, if subjects were certain about the letter they report but uncertain about its position relative to the cue, they will give low confidence ratings (knowing that the letter may not be from the cued position) but they will still be correct on many trials. This (location uncertainty) could thus explain the increased performance for short ISIs at low confidence.

To disentangle these two possibilities, we performed another experiment in which subjects were asked to report their confidence on two different dimensions – letter identity and position of the seen letter relative to the cue - in two successive screens [see methods, Dual-Confidence Experiment]. We then represented this data in a scatter plot of position vs. target-identity confidence (Figure 2E–F). As in our previous result we observed that even for correct responses, the confidence rating showed a broad dispersion and were not clustered at high confidence ratings. In correct trials we observed many instances in which subjects showed high confidence for the position but not for letter-identity, and very rarely the converse relation, i.e. trials in which subjects were certain about the target they saw, but uncertain about their position relative to the cue (Figure 2E). This was reversed in error trials, where we observed that the distribution was biased towards certainty about the reported letter but uncertain about its position relative to the cue (Figure 2F). While not conclusive, this data suggests that the low-confidence correct responses result from the absence of awareness of target identity and thus relate to blindsight, suggesting a short-time scale for this phenomenon.

In the next section we address the spatial specificity of this bias, investigating the relation between the precise location of the cue, of the responded letter and of the subjective rating of confidence.

### 4- Spatial biases: the spatial maps of forced-choice responses and subjective confidence

**Figure 3:**
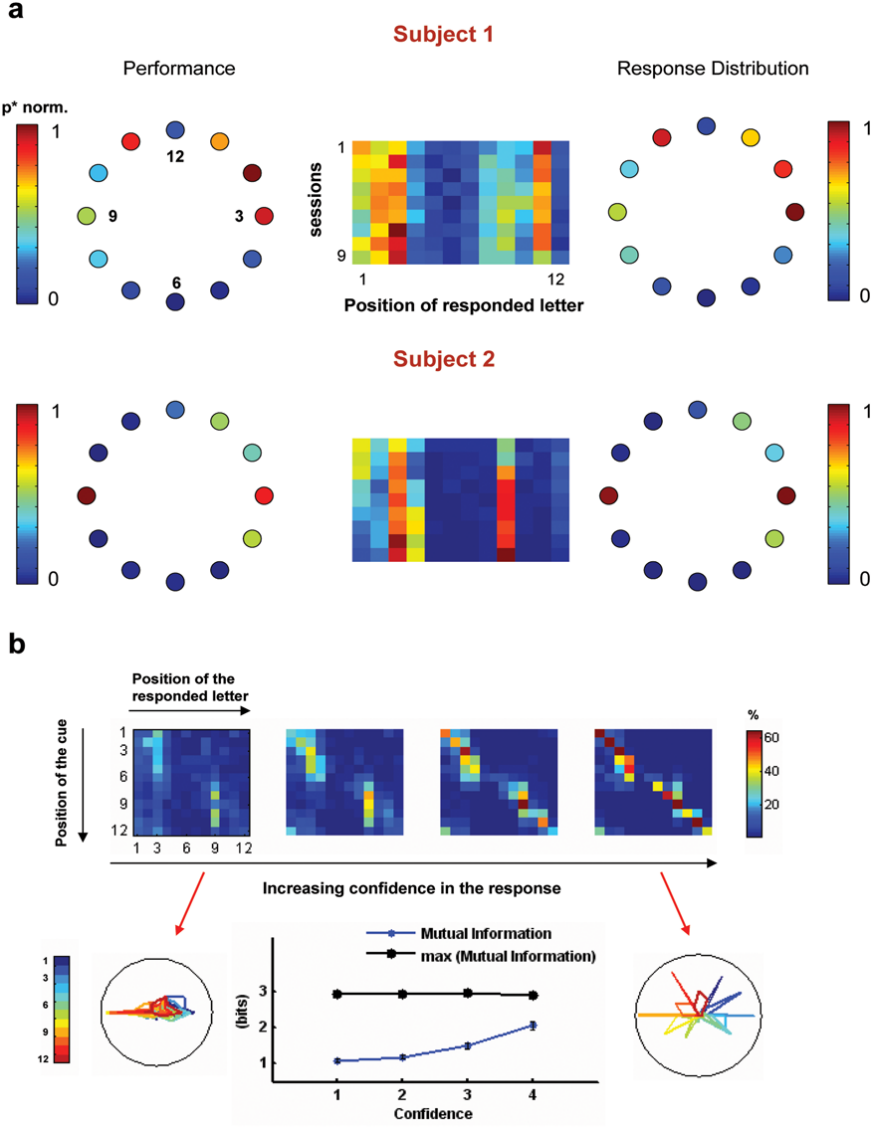
Spatial biases in response distributions and subjective confidence. A) Left panels: Mean performance (p*) across positions for each subject. Center and Rigth panels: Probability of responding to a specific position in the array (independently of the position of the cue). Center: Each column corresponds to a location in the array (Nth column refers to N o'clock position. Each line corresponds to a different session. Right: Data collapsed across all sessions. B) Top Panel: Probability of responding to the location (i, in columns) given that the cue was in location (j, in lines), P(i,j). The matrix P approaches the diagonal (correct responses) for increasing levels of subjective confidence. Bottom panel: P in a polar representation for the lowest and highest subjective confidence. Mutual information of the distributions of the position of the cue and of responded letters increases with confidence but does not saturate even for the highest confidence values.

As found in previous studies, here we observed consistent differences in performance as a function of the position of the cue, even in the absence of any task-related positions specificity, since the cue appeared with equal probability in all locations [14]. The left panels of Figure 3A show the performance for each individual subject as a function of the location within the array, grouping the data across all ISI values and letter identities. This bias in performance was largely determined by a bias in the response – as indicated by the maps of the position of the responded letter (regardless of the position of the cue) which were very heavily weighted towards certain specific positions of the array (Figure 3A, right panels). To assess the reliability of this measurement, and to investigate possible mechanisms which may lead to the spatial inhomogeneity in performance, we explored the variability of the spatial maps of responded letter for each individual subject in different experimental sessions (Figure 3A, center panels). These results showed that, for each subject, the pattern of responses showed consistent regions with very high probability of response (for instance participant 2 has a very strong bias to report the stimulus presented at 3 and 9 o'clock). We also found several positions which were virtually “blind” to both participants, particularly in the lower hemi-field. This response bias was remarkably stable as can be seen by the analysis of responses for different experimental sessions, each performed in a different day. Importantly, the position bias seems to be completely unconscious as participants were unaware of the fact that they were responding with unusually high probability to letters in specific locations of the array.

This finding suggests that for a substantial amount of trials subjects response is virtually independent of the position of the cue, yet highly predictable due to an unconscious spatial bias in the response. To understand whether the subjective confidence score may identify these trials, we investigated the precise probability of response for different positions of the cue and values of subjective confidence. We measured, for each confidence level, the stimulus response matrix *P(Ri,Sj)*, where *P(i,j)* is determined by the probability of responding the letter in position i (Ri) given that the cue was in position j (Sj). The matrices for different levels of subjective confidence are plotted, from left to right, in Figure 3B. The lines represent the position of the cue and columns the position of the responded letter. In this representation, elements in the diagonal correspond to correct responses, elements close to the diagonal to approximately correct responses and elements far from the diagonal to error trials. The pattern for low confidence value responses is very interesting: two vertical segments corresponding to the right most location of the vertical meridian (column 3) which is responded when the cue was presented in the right (lines 1 to 5) and the left-most location of the vertical meridian (column 9) which is responded when the cue was presented in the left (lines 7 to 11).

Quantitatively, this is reflected in the fact that – for low subjective confidence values – the mutual information between the stimulus and the response is 1 bit. This essentially signifies that from a low-confidence report, an observer can determine whether the position of the cue belongs to one of two categories (the right or left hemi-field) from the participants' response. The mutual information increases substantially with the subjective confidence report (ANOVA, F_3, 3_ = 14.29, P = 0.0275) but even at high levels of confidence it reaches value close to 2 bits indicating that, on average, the “resolution” of a high confidence report is of about three positions (67° of the array). This can be seen qualitatively from the stimulus response matrix at high confidence subjective ratings which shows responses packed close to the diagonal, with a variability which varies between 2 to 4 positions. This result is in line with our previous finding of the existence of high confidence errors which involve responses of elements of the array which were close to the cued location.

### 5- The (short) temporal evolution of confidence

In the previous sections we showed evidence that the temporal fading of information was distinct for objective performance and subjective confidence rating. This finding could be related to interactions between spatial, temporal and error rate variables. In this last section, we study explicitly the evolution of confidence in time as the other experimental factors are maintained fixed.

**Figure 4:**
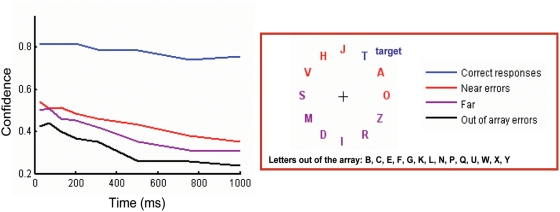
The temporal evolution of confidence. Time course of subjective confidence responses after separating responses in correct (blue), near (red) and far (purple) errors (in which the distance between the target and the cue was smaller/larger than three), and errors in which the responded letter was neither the target nor the distractor (black). The subjective confidence decreases with time when spatial factors are factored out.

We first analyzed the evolution of subjective confidence ratings as a function of ISI, which shows a monotonic decrease. To investigate whether there is a pure effect of time (independent of the other factors which covariate with ISI) in the estimate of confidence, we measured the mean confidence rate for correct responses, and for trials with errors either proximal to the target (d<3) or far from the target (d>3) or errors in which the responded letter was not one of the distractors (Figure 4). The function of confidence as a function of ISI for error trials are roughly parallel indicating that there is a main effect of ISI but without interaction between the different error types (ANOVA, main effect of ISI, F_7, 7_ = 5.52, P = 0.0193; main effect of error type, F_2, 1.94_ = 15.14, P = 0.0657; ISI * Error Type, F_14, 309_ = 0.89, P = 0.5718).

This suggests that, independently of all other factors, there is a monotonic, roughly exponential, decay of subjective confidence during the few hundred milliseconds between stimulus presentation and the response.

## Discussion

Renewed attention from the neuroscience community has been directed to quantitative studies of introspection, the measurement of subjective confidence and its relation to objective measurements [18]–[20] [8], [21], [22]. In this study we used a partial report paradigm, combined with an introspective report, to investigate, in a quantitative manner, the spatial and temporal factors determining the construction of subjective confidence reports. Our main findings are: 1) Objective performance beyond explicit subjective reports (blindsight) decreases substantially after short – less than 500 ms – temporal intervals. 2) The reversed situation of high confidence errors in which subjects believe in an objectively incorrect response were largely determined by spatial proximity. 3) Low confidence errors were highly structured: responses were heavily weighted to certain specific locations of the visual field, which were very reliable within each subject for different sessions and 4) Subjective confidence showed a moderate but consistent decrease with time, independent of all other experimental factors.

### Performance in a world of low subjective confidence: The spatial and temporal windows of unconscious priming

As in many other studies, we found in this study evidence for correct responses with low subjective confidence [5], [10], [21], [23]. The dual confidence report experiment further suggests that this results from absence of awareness of target identity, thus constituting an example of blindsight. Our results then suggest that the ability to rout information from the sensory to the decision making machinery in the absence of consciousness decreases substantially after a few hundred milliseconds.

This is consistent with other lines of investigation predominantly from the priming literature [24]–[26], from the trace and delay operational conditional learning [27], [28] and with theoretical models of consciousness which argue that an important aspect of consciousness it to maintain and broadcast information flexibly across modalities, time and space [19], [29], [30]. Some studies however suggest that unconscious primes may act on a less flexible manner for longer durations. First, in priming experiments exponential effects have been observed as a recurrent influence of past trials on present behavior (inter-trial perserveration) in normal subjects [31] as well as in brain-lesioned patients [32]. Second, in a study combining a partial report with a change blindness paradigm, it was found that the capacity measure (of deciding the orientation of a rectangle) was between 6 and 7 items even at about 1.5 seconds after stimulus presentation [33]. A theoretical argument sustains that during this period subjects maintain a rich and detailed phenomenal representation of the visual display which is only partially accessible for report and thus cannot be measured with our methodology [18]. Indeed, our results can only reflect the accessible elements of consciousness and hence our conclusions are agnostic to the existence of a richer phenomenological internal construction.

Our results also suggest that in the low subjective confidence responses the response was heavily determined by stimuli presented at very precise locations within the visual field. The most interesting aspect of this observation is that subjects where completely unaware of this fact, i.e. they did not report a conscious strategy of reporting the sole letter of the array which they had seen, even if it was not in the cued location. This suggests a speculative but theoretically interesting line of thought: 1) the “chance” response is strongly conditioned by a prime, determined by the letters that were contained in the array in the uncued location (distractors) and 2) that the probability that a distractor will act as a response prime is strongly determined by its spatial location in the array. The first aspect of these observations presents no surprise. An enormous number of reports, in different circumstances have indicated that what appears to be a “random” response to a subject is conditioned by a previous unnoticed event [14], [23], [24], [34], [35]. The second aspect is the most interesting one since it suggests a very uneven weighting of the “priming efficiency” of twelve letters presented at different angles of the visual field. This distribution was indeed very reliable from session to session within each subject and, showed consistency across subjects: both showed a strong effect in the horizontal meridian and a stronger tendency towards the right visual field, as expected due to spatial allocation reading bias [36]–[38].

### Correct and quasi-correct performance in a world of high subjective confidence: The spatial resolution of conscious report

A large literature has also addressed the construction of high-confidence errors, mostly in relation to the creation of false memories. More related to this study, high-confidence errors have been shown to increase in cluttered fields, as demonstrated in an experiment in which subjects determined the magnitude and direction of the tilt of a target grating [39]. In our experiment we could, as discussed previously, understand the nature of the errors that result in high-confidence responses.

We observed that *high-confidence errors* involved mostly responses of letters adjacent to the cued location and the distribution of distance reached a plateau at a distance of three elements. Since most of the observed variables in this experiment showed an effect of ISI, we explored whether this distribution changed for long and short ISI values. Interestingly, this distribution did not depend on ISI, indicating that the misattribution in space does not result from a progressive drift and loss of spatial resolution but, more likely, to a coarse spatial access determined by the cue. Other experiments have reported a related phenomenon, indicating that the visual system can be correctly informed about target *presence*, yet be misled about its actual *location* by constraints [40]–[42]. While in these studies subjective confidence was not measured explicitly, in most of these studies it is reported informally that subjects have a very vivid perception of the illusory location. Also, in most of these studies, the misattribution of location was interpreted in different variants of “most-likelihood” estimates determined either by priors or by geometrical constraints of the visual field. An interesting aspect of this study is that the cue may act as an attractor of a broader region in space, and within this relatively coarse kernel, spatial precision is lost and thus subjects construct a high confidence estimate of their response.

Previous studies on partial report task reported – as in this study – a high fraction of localization errors. These previous studies have used linear and for the most part smaller arrays [43]–[46] and thus localization errors have been interpreted in terms of a foveal bias, an incorrect localization of the cue or simply “guessing” in a small subset [17], [47], [48]. In our design, in which all targets were presented at equal eccentricity, with less likelihood of crowding and with an easy labeling of the cued location during all the decision process (the cue was present during that time and subjects could report without confusion the position of the cue) this rationale cannot explain localization errors. We tentatively suggest that these localization errors – which were accompanied by a high subjective confidence in the response and confined to a short window of proximity to the cue - reflect intrinsic spatial limits on the attentional resolution system and the allocation of top-down control [49]–[51].

## Materials and Methods

### Participants

Two native Spanish speakers (1 male, 1 female) with an age of 24 and 27 years olds participated in this experiment. Both participants reported normal or corrected-to-normal vision, and both were graduate students from Faculty of Exacts and Natural Science, University of Buenos Aires (Buenos Aires, Argentina). All participants gave written consent to participate in this study. One of the participants MG – was an author.

### Visual Stimuli and Procedure

Behavioral experiments were programmed in Python (www.python.org). In each trial, twelve letters were presented simultaneously for 106 ms (corresponding to 9 frames with a refresh rate of 85 Hz) on the screen following 600 ms of fixation. Stimuli were presented on a 19″ screen (resolution of 800×600 pixels) placed at a distance of 73 cm. Letters fonts were uppercase Time New Romans with a size of 1.2°. Letters were chosen randomly from the alphabet (26 symbols), without repetition. The twelve letters were arranged on a circle, around the fixation point at an eccentricity of 5.2°. A red dot (0.1°) on an array of blue dots (with the same configuration of the letters but at an eccentricity of 5.5°) indicated the position of the target. Participants were asked to report, using a standard keyboard, the letter presented in the position cued by the red circle, which remains on screen until subject's response. Subsequently, participants had to report the confidence of their response with and *ad hoc* bar placed in the center of the screen and composed of 13 marks and two labels: “0% Confidence” and “100% Confidence” (“0% Seguro” and “100% Seguro”, in Spanish). The participants could move freely the mouse to select the appropriated response. Eight Inter-Stimulus-Intervals (ISI) were used (24, 71, 129, 200, 306, 506, 753 and 1000 ms). In all conditions, the cued stayed on the screen until the subjective report bar was presented.

Each observer first completed a practice block of 96 trials before the first session. In subsequent sessions, the practice block was reduced to 30 trials. The participants that participated in this study had previously be part of other similar experiment [14] and had extensive practice in psychophysics experiments.

Subjects completed 9 sessions of 6 blocks each one (576 trials for session). In each block all positions (total 12) and all ISIs (total of 8) were randomly and uniformly sampled. Participants were instructed to fixate in the center of the screen during the entire experiment and to report the letter as fast as they could, within a forced-choice between the 26 letters of the alphabet. Each session lasted approximately 45 minutes and was performed in different days.

We performed four control experiments which were different variants of the main experiment described above. Each control experiment involved a single session. The same participants which completed the main experiment performed, in following sessions the control experiments:

[Cue Position Control Experiment]

Participants performed 120 trials. The stimulus display was exactly as in the original experiment. After completion of the trial, the screen disappeared and after 1 sec, the subject was shown an array with all the locations and asked to report position of the cue.

[Feedback Control Experiment]

Participants performed 576 trials, as in a regular session of the main experiment. In error trials in which the distance was smaller than 3, subjects were informed with a single tone that the trial was incorrect. In a subsequent screen they were asked, in a two-forced choice (responded with the index and middle finger of the right hand) to report whether the responded letter was clockwise or anticlockwise relative to the cue.

[Dot Probe Experiment]

Participants preformed 120 trials. The design was identical to the main experiment, except that during the display of the letters a small dot probe was presented on the inside of the ring of letters. In a subsequent screen, following the trial, subjects were asked to report (as in the previous experiment) whether the dot was clockwise or anti-clockwise respect to the cue.

[Dual-Confidence Experiment].

Participants preformed 576 trials. The design was identical to the main experiment, except that participants responded to two consecutive subjective confidence estimates. They first indicated the confidence that the responded letter was the letter they had seen, and then the confidence that the responded letter was in the position indicated by the cue.

### Data Analysis

We conducted a longitudinal experiment in which we measured performance for participants during repeated sessions (9 sessions). All individual sessions showed a consistent decay of performance with ISI, indicating that participants acquired stable performance across sessions.

Performance data were corrected by false positives (FP), using the following equation
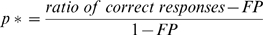



FP defined as the response probability for a specific letter given that it was not presented as the target. FP were calculated for each individual letter, independent of the ISI value. FP were bellow 3% for all conditions and thus corrected performance was not substantially different than the non-corrected performance. Introspection data for each session was normalized between 0 and 1 across session and participants.

#### Statistics

tatistics were done through Analysis of Variance (ANOVA) with subjects and sessions as random effects, and assuming a normal distribution for the data. *Post hoc* analyses were done through Bonferroni Test. In all tests, alpha level was 0.05.

#### Mutual Information Analysis

Mutual information was calculated -for each confidence category and subject- through the following equation:
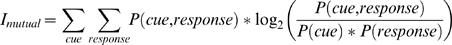
where P(cue) was the probability of target appearance in a specific position (as the experiment is balanced, this probability is the same for all target positions), P(response) is the probability to response with a letter in a specific location in the array (i.e., the probability to response a letter in position 1, independent of cue position), and P(cue, response) is the probability to response a letter in a specific position, with the cue presented at the same or a different (but specific) location. The maximum value of Mutual Information (as the limit of information that could be transmitted) for each confidence category and subject was calculated as the minimum value between the entropy of cue position and the entropy of response position distribution.
